# A Unique Dual Activity Amino Acid Hydroxylase in *Toxoplasma gondii*

**DOI:** 10.1371/journal.pone.0004801

**Published:** 2009-03-11

**Authors:** Elizabeth A. Gaskell, Judith E. Smith, John W. Pinney, Dave R. Westhead, Glenn A. McConkey

**Affiliations:** 1 Institute of Integrative and Comparative Biology, University of Leeds, Leeds, United Kingdom; 2 Institute of Molecular and Cellular Biology, University of Leeds, Leeds, United Kingdom; 3 Faculty of Life Sciences, University of Manchester, Manchester, United Kingdom; Instituto Butantan, Brazil

## Abstract

The genome of the protozoan parasite *Toxoplasma gondii* was found to contain two genes encoding tyrosine hydroxylase; that produces l-DOPA. The encoded enzymes metabolize phenylalanine as well as tyrosine with substrate preference for tyrosine. Thus the enzymes catabolize phenylalanine to tyrosine and tyrosine to l-DOPA. The catalytic domain descriptive of this class of enzymes is conserved with the parasite enzyme and exhibits similar kinetic properties to metazoan tyrosine hydroxylases, but contains a unique N-terminal extension with a signal sequence motif. One of the genes, TgAaaH1, is constitutively expressed while the other gene, TgAaaH2, is induced during formation of the bradyzoites of the cyst stages of the life cycle. This is the first description of an aromatic amino acid hydroxylase in an apicomplexan parasite. Extensive searching of apicomplexan genome sequences revealed an ortholog in *Neospora caninum* but not in *Eimeria*, *Cryptosporidium*, *Theileria*, or *Plasmodium*. Possible role(s) of these bi-functional enzymes during host infection are discussed.

## Introduction

*Toxoplasma gondii* is among the most successful of parasites with the potential to infect all warm blooded animals and with an estimated prevalence of 30% in the world's human population (1,2). Infection normally consists of an acute stage in which the rapidly growing tachyzoites infect a range of tissues, followed by a latent stage during which slowly replicating bradyzoites form tissue cysts in muscle and brain (3,4). The tropism of parasites for brain tissue is intriguing and has been linked with specific behavioural changes. In rodents infection with *T. gondii* has been shown to modify aversion to predators that might facilitate transmission of parasites (5,6). There are also reports indicating infection may lead to psychological sequelae in humans. The mechanism responsible for the behavioural changes remains unclear.

As an intracellular parasite Toxoplasma is known to remodel host cellular processes to supply the nutrients required for parasite growth, protect against host defences, and facilitate replication and transmission. During invasion and formation of the parasitophorous vacuole, the parasite modifies the host cytoskeleton through reorganization of intermediate filaments and capture of microtubule organization centers and forms associations with mitochondria and ER. The complement of host proteins changes during tachyzoite infection with the induction of numerous mitochondrial and several ER proteins (9–11). Subversion of the host cell modifies transcription (e.g. ROP16 modulating STAT), blocks apoptosis (e.g. NF-kappaB, caspases) and ensures supply of essential nutrients (e.g. purines, polyamines, and cholesterol). *T. gondii* relies on a combination of synthesis and salvage for supply of the various amino acids. For example the parasites are auxotrophic for tryptophan and arginine (12–14).

In the current study, we screened the *T. gondii* genome using metabolic pathway reconstruction software (15–17) to identify potentially secreted enzymes. This led to the unexpected identification of an aromatic amino acid hydroxylase, a gene of interest due to its potential to synthesise the signalling molecule l-DOPA (3,4-dihydroxy-l-phenylalanine). We here describe cloning expression and functional ratification of this molecule.

## Results

### Identification of a tyrosine hydroxylase with a signal peptide sequence in *T. gondii*

**Figure 1:**
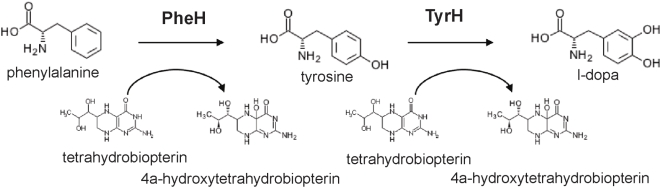
Schematic of the reactions catalyzed by tyrosine and phenylalanine hydroxylases. Tyrosine hydroxylase catalyzes the conversion of tyrosine to l-DOPA and phenylalanine hydroxylase catalyzes the conversion of phenylalanine to tyrosine. Tyrosine hydroxylase is the rate limiting step in the synthesis of the catecholamines. The tetrahydrobiopterin cofactor, essential for the activity of these enzymes, is metabolized to 4a-hydroxytetrahydrobiopterin during the hydroxylation of the amino acid.

To identify metabolic enzymes that may be secreted from *T. gondii* into its host cell, we compared the enzyme set resulting from applying the SHARKhunt search algorithm to the *T. gondii* genome to the set of proteins at ToxoDB with predicted signal peptides (www.toxodb.org (18,19). A list of 106 enzymes was identified. Sequences with similarity to signal peptides have been found in proteins trafficked to the parasite organelles (e.g. dense granules, apicoplast, mitochondria) as well as secreted out of the parasite. The known organelle proteins in the mitochondria, apicoplast, and micronemes were removed from the list. Among the classes of enzymes remaining, the phosphatases may be targeted to the acidicalcisomes and UDP-N-acetylglucosamine utilizing enzymes may be lysosomal. Several of the enzymes (six) are involved in post-translational modification and may be localized to the ER or Golgi apparatus. Sets of kinases and proteases were found that may be secreted or organellar (e.g. rhoptry, micronemal), although their specific annotation is complicated by their membership in large gene families. Among the remainders we focused on an unexpected metabolic enzyme with a predicted signal peptide but no predicted transmembrane helix; a predicted tyrosine hydroxylase. Surprisingly, there were two hits for tyrosine hydroxylase in predicted genes in the genome of *T. gondii*. Tyrosine hydroxylases have not previously been described in protozoa but are only found in animals where they form the rate-limiting step in synthesis of dopamine (Fig 1). Tyrosine hydroxylase is a member of a highly conserved family of aromatic amino acid hydroxylases consisting of tyrosine, phenylalanine, and tryptophan hydroxylase. Phenylalanine hydroxylase catabolizes phenylalanine to tyrosine and tryptophan hydroxylase catabolizes tryptophan to 5-hydroxytryptophan. All described aromatic amino acid hydroxylases are cytosolic and there are no detectable signal peptide sequences (based on SignalP analysis of GenBank entries, data not shown). Due to its presence in a parasitic protozoan and the unique signal peptide prediction we were interested to further characterize this enzyme.

We performed sensitive bioinformatic searches of genomes of parasitic protozoa in the crown group alveolates whose sequences are currently publicly available. Among the apicomplexan genomes searched by SHARKhunt (*P. falciparum*, *P. chabaudi*, *P. berghei*, *C. parvum*, *C. hominis*, *B. bigemina*, *T. annulata*, *T. parva. N. caninum*, and *E. tenella*), the only hit for a tyrosine hydroxylase gene or any aromatic amino acid hydroxylase was in *N. caninum* (E-value of 4.0×10^−99^) and no hits (E-value<1×10^−10^) in any of the other apicomplexan genomes analysed.

### *T. gondii* contains two genes encoding tyrosine hydroxylase

**Figure 2:**
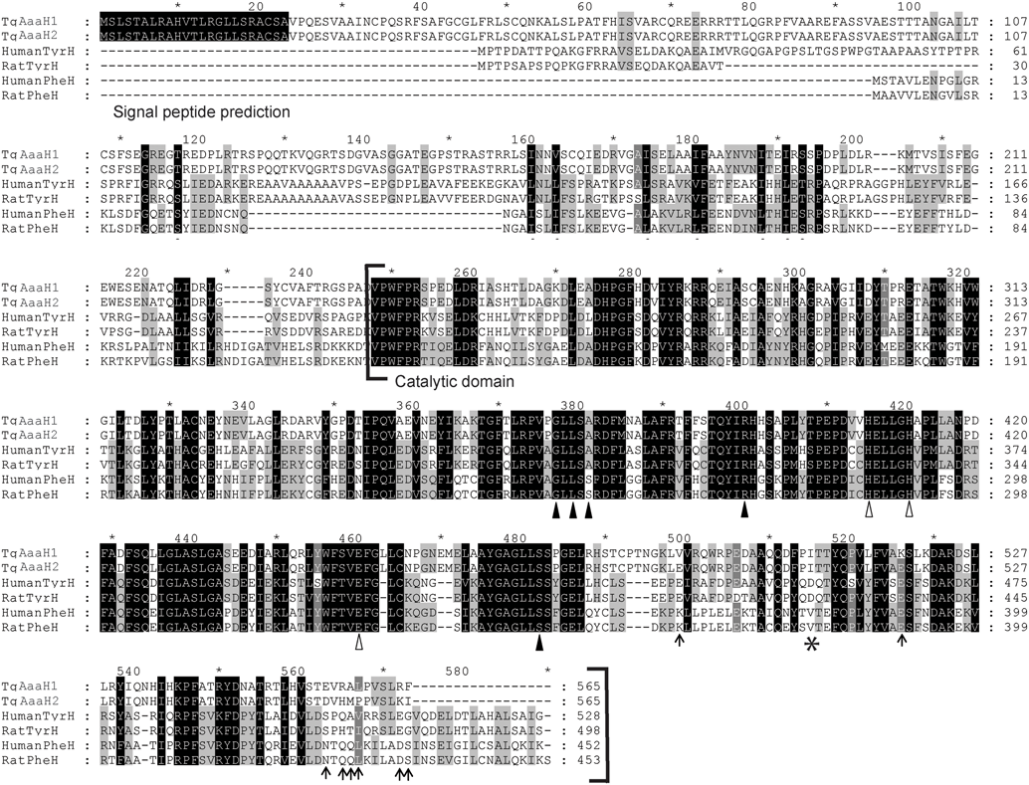
Alignment of aromatic amino acid hydroxylases. Aromatic amino acid hydroxylase sequences from the genes of *T. gondii* (GenBank™ accession TgAaaH1 – EU481509, TgAaaH2 – EU481510) human TyrH isoform 4 (AAA61179), rat TyrH isoform 1 (AAA42257), human PheH (P00439) and rat PheH (AAA41843). The alignment was generated using MUSCLE alignment software [26]. Identical residues are highlighted in black. The *T. gondii* proteins appear more similar to mammalian TyrH isoform 4 than isoform 1 [43]. The iron binding residues are highlighted with open triangles. The residues present in the active site that interact with the aromatic amino acid substrate and biopterin cofactor are highlighted with black triangles. The conserved residue implicated in substrate specificity is highlighted with an asterix; D425 encoded by rat TyrH and V379 of rat PheH. Residues that differ between the two *T. gondii* proteins are highlighted with arrows. The predicted N-terminal signal peptide based on a SignalP (59) prediction is also highlighted. The conserved VPWFPR motif identifies the start of the catalytic domain shown in brackets (61).

Two nearly identical genes were identified in the *T. gondii* genome with similarity to metazoan tyrosine hydroxylase. They correspond to gene models 77.m00053 and 31.m00940 in the *T. gondii* genome at ToxoDB (www.toxodb.org). The sequences were also found in EST libraries for tachyzoites and bradyzoites (www.eupathdb.org). The two genes are located on chromosome V separated by ∼450 Kbp (kilobase pairs) with identical introns with the exception of a few nucleotide differences in intron 7, however the 5′ and 3′ UTR regions do not contain any similarity. The 5′ and 3′ ends of the transcript were mapped by overlapping PCR and 3′ RACE. This verified the coding sequence and placement of the initiation and stop codons. These sequences were submitted to GenBank as TgAaaH1 and TgAaaH2 (*Toxoplasma gondii* aromatic amino acid hydroxylase) and given the identifiers EU481509 and EU481510, respectively. The coding sequences of two genes encode proteins of 565 residues and are 98.6% identical (Fig. 2). The paralogous *T. gondii* proteins differ by eight residues in the C-termini.

The *Toxoplasma* genes share high levels of similarity in the catalytic domain to mammalian tyrosine and phenylalanine hydroxylases. Alignment with the rat and human enzymes (Fig. 2) reveals a clear region of similarity that begins at residue V164 of rat tyrosine hydroxylase and V240 of the *T. gondii* sequences (20). This residue denotes the beginning of the catalytic domain with the conserved VPWFPR motif found in all tyrosine and phenylalanine hydroxylases (21,22). The residues that interact with the biopterin cofactor and amino acid substrates in the rat TyrH (tyrosine hydroxylase) sequence (23) are denoted by closed triangles in Fig. 2. The catalytic domain also contains the conserved iron binding residues at positions His407, His412 and Glu452 corresponding to His331, His336 and Glu376 in rat and human tyrosine hydroxylase (24), shown highlighted with open triangles in Fig. 2. Modelling of the structure of the *T. gondii* catalytic domains on the crystal structure of rat tyrosine hydroxylase corroborates the sequence alignment and placement of key residues (data not shown). The N-terminal domain, which is associated with regulation in other eukaryotic aromatic amino acid hydroxylases, is less conserved than the catalytic domain (20). The N-termini of the two *T. gondii* enzymes contains a predicted signal peptide as expected from the original bioinformatics search (Fig. 2). This is also found in the predicted *N. caninum* gene. This is the only example of a tyrosine or phenylalanine hydroxylase with a signal peptide. Hence, this normally cytosolic enzyme may be secreted in this intracellular parasite.

**Figure 3:**
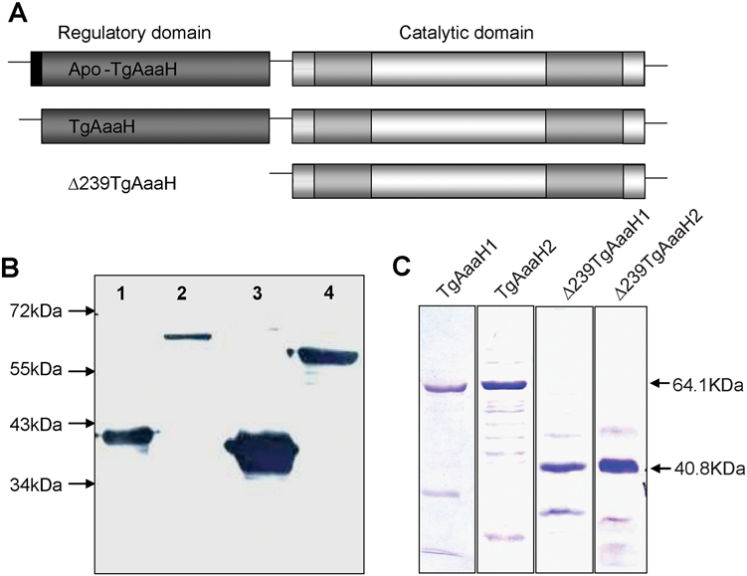
*T. gondii* aromatic amino acid hydroxylases expressed in this study. A Schematic representation of three forms of TgAaaH 1&2 expressed in this study. Apo-TgAaaH 1&2 encode the full length wildtype protein expressed in preliminary trials as a 6×His-recombinant protein of 601 amino acids (a.a.) and 66.5 kilodaltons (kDa), with the predicted signal peptide shown in black. As this protein was found to be extremely insoluble, the N-terminal 23 codons containing the predicted signal peptide was removed generating constructs named TgAaaH 1&2. The resulting proteins expressed are 578 a.a. and 64.1 kDa. Δ239TgAaaH 1&2 have the N-terminal 239 a.a. deleted resulting in expression of proteins of 361 a.a. and 40.8 kDa. B Western blot of proteins expressed from Δ239TgAaaH2 and TgAaaH2 and the recombinant rat protein controls probed with anti-rat tyrosine hydroxylase. *Lane 1*, Δ239TgAaaH2, *lane 2* TgAaaH2, *lane 3* rat-Δ117PheH and *lane 4* rat-WT-TyrH. Due to the high degree of similarity with the rat enzyme, recombinant Toxoplasma proteins are recognized by antibody raised against the conserved domain of the rat tyrosine hydroxylase enzyme. C Coomasie stained SDS-PAGE gels of purified, recombinant proteins encoded by TgAaaH1, TgAaaH2, Δ239TgAaaH1 and Δ239TgAaaH2.

**Figure 4:**
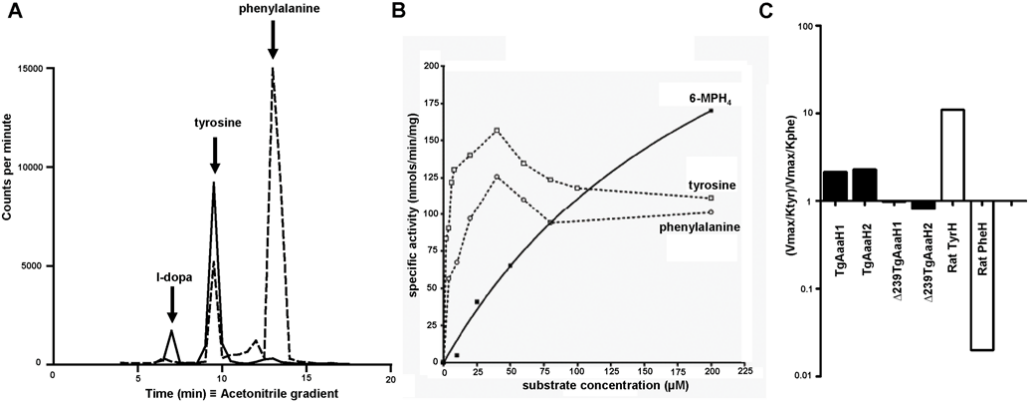
TgAaaH 1&2 encode tyrosine hydroxylase with unusual substrate specificity. A HPLC tracing of production of tyrosine and l-DOPA by the recombinant enzymes. Fractions were collected and radiolabel monitored by scintillation counting. Internal markers of phenylalanine, tyrosine, and l-DOPA are marked by arrows. B Graph showing the catalysis of tyrosine and phenylalanine in relation to substrate concentration. The substrates are tyrosine (0–200 µM) and phenylalanine (0–200 µM). The dependency on the tetrahydrobiopterin analog 6-MPH_4_ over a range of concentrations is also shown with tyrosine (50 µM) as substrate. C Relative catalysis of tyrosine and phenylalanine reveal substrate preference. Reaction rates and K_M_ of the substrates were measured and the ratios are shown. A ratio of 1 indicates no substrate specificity. A ratio of less than 1 indicates a preference for phenylalanine as substrate; demonstrated by the inclusion of the rat phenylalanine hydroxylase (20). A ratio of greater than 1 indicates a preference for tyrosine as demonstrated by the inclusion of rat tyrosine hydroxylase.

To demonstrate activity, recombinant proteins of the two *T. gondii* genes were expressed as N-terminal His-tagged fusion proteins under control of the T7 promoter (Fig. 3). Yields of both genes were ∼0.75 mg/L culture of recombinant proteins with ∼80% purity. Removal of the N-terminal signal peptide resulted in an ∼5-fold increase in the yield of soluble protein. Tyrosine, phenylalanine and tryptophan were tested as substrates in enzyme assays with the recombinant enzymes. There was no detectable activity of the *T. gondii* enzymes with tryptophan, but the enzymes were able to catabolize tyrosine and phenylalanine. Assays were performed with ^3^H-tyrosine and ^3^H-phenylalanine as substrates and the products analyzed by HPLC (Fig. 4A). A peak was detected comigrating with l-DOPA was observed with tyrosine as a substrate demonstrating the tyrosine hydroxylase activity. A peak comigrating with tyrosine was observed with phenylalanine as a substrate. This correlates with results of a coupled assay (25,26) that shows catalysis in a dose-dependent fashion with tyrosine and phenylalanine as substrates (Fig. 4B). The biochemical parameters of the recombinant enzymes were measured (Table 1). The paralogs behave similarly in all biochemical assays. The parasite enzyme has a lower K_M_ for tyrosine than rat tyrosine hydroxylase. The K_M_ for phenylalanine was 2.45–14.8 µM; considerably higher than rat phenylalanine hydroxylase. Hence, the *T. gondii* enzyme is bifunctional. Interestingly, as found for other members of this family, the reaction is substrate-inhibited at high substrate concentrations (Fig. 4B).

**Table 1:** Steady state kinetic parameters for tyrosine and phenylalanine hydroxylation by *T. gondii* aromatic amino acid hydroxylases.

	K_M(tyr) (µM)_	V_max(tyr) (nmol/min/mg)_	K_M(phe) (µM)_	V_max(phe) (nmol/min/mg)_
TgAaaH1	1.25±0.69^a^	137±14^a^	2.45±1.87^a^	125±15^a^
TgAaaH2	5.50±3.26^a^	123±1^a^	14.8±6.4^a^	145±37^a^
Rat TyrH	16±3^b^	150±14^b^	109±20^b^	93±12^b^
Rat PheH	>200^b^	nd^b^	288±59^b^	373±21^b^

a Conditions: Varied concentrations of 0–200 µM tyrosine or phenylalanine, 50 mM HEPES-NaOH, 10 µM ferrous ammonium sulphate, 500 µM NADH and 500 µM 6-MPH_4_ (pH 7.5 and 32°C). The error given is the SEM of two or three separate experiments performed in duplicate or triplicate.

b Data taken from (20), oxidation. The activity PheH with tyrosine is so low that an accurate value could not be measured due to extremely low activity. nd = not detected.

To assess the relative substrate specificity of each *T. gondii* enzyme, the V_max_/K_m_ for tyrosine and phenylalanine was compared as in previous publications (27). We reproduced experiments with recombinant rat tyrosine and phenylalanine hydroxylases that demonstrate substrate specificity (20). The rat enzymes have strict specificity, with phenylalanine having greater than 100-fold preference for phenylalanine over tyrosine (Fig. 4C). The *T. gondii* enzymes have a two to three-fold preference for tyrosine and are therefore more similar to tyrosine hydroxylases. In keeping with previous naming systems based on substrate preference, the *T. gondii* enzyme is named a tyrosine hydroxylase. The *T. gondii* enzyme differs from other tyrosine hydroxylases in its catalytic efficiency with both substrates and is, hence, bifunctional.

Aromatic amino acid hydroxylases are dependent upon the cofactor tetrahydrobiopterin for activity. The recombinant *T. gondii* enzymes exhibited an absolute requirement for biopterin for activity using the stable tetrahydrobiopterin analog 6-MPH_4_ (6-methyl-tetrahydropterin) At concentrations <20 µM 6-MPH_4_ no catalysis was observed. A dose-response curve of activity was obtained over a range of biopterin concentrations (Fig. 4B). The differences in the curves for substrate and cofactor is similar to other tyrosine hydroxylases and is likely to indicative of the biochemical mechanism of catalysis as discussed below (25). The K_M_ of 6-MPH_4_ for the enzymes was 310–318 µM with specific activities of 123–137 nmol/min/mg. These are similar to the values observed for rat tyrosine hydroxylase.

To determine the location of the catalytic domain and its contribution to substrate specificity, deletion mutants of both genes were generated. The N-terminal 238 residues were deleted to the conserved VPWFPR (Fig. 2). The activity of these mutant enzymes (Δ239TgAaaH1&2) was assayed for comparison to full length enzymes. There was no loss of catalytic activity in the mutants. The mutant enzymes had K_M_ values for tyrosine of 3.22±1.15 and 4.12±0.10 µM and V_max_ of 185±10 and 148±42 nmol/min/mg, respectively. Hence the C-terminal sequence following residue 239 is sufficient for catalysis. The deletion mutants were examined to determine whether the catalytic domain also provides the substrate specificity. The catalytic domain only mutants have lost their preference for tyrosine (Fig. 4C). Hence the N-terminal sequence is responsible for the tyrosine specificity.

### Expression of the *T. gondii* hydroxylase is regulated through development

**Figure 5:**
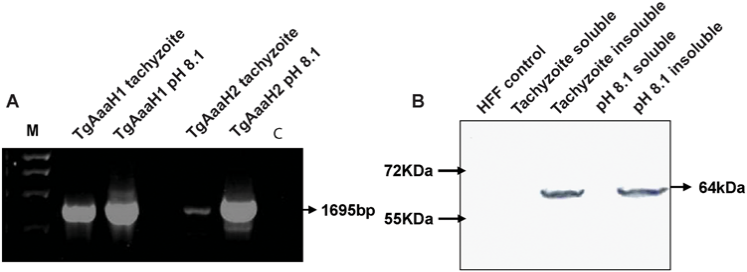
Expression of *T. gondii* tyrosine hydroxylases in tachyzoites and bradyzoites. A 1% Agarose gel showing detection of RT-PCR products with TgAaaH 1&2 specific primers from tachyzoites and 5 day culture induced bradyzoites from NED strain parasites. *M* is the DNA marker and *C* is a no-template control. All products are the correct size for the ORF's minus the introns. B Western blot of extracts of Type III NED strain tachyzoites, induced bradyzoites (5 day) and HFF extract negative control probed with anti-rat-tyrosine hydroxylase antibody. A band of 64 kDa corresponding to the predicted size of *T. gondii* tyrosine hydroxylase can be seen in both tachyzoite and bradyzoite insoluble fractions. Due to the high level of similarity between these two proteins it was not possible to differentiate between them.

We examined the *in vivo* expression of the tyrosine hydroxylase. Initially, parasite cultures containing a mixture of tachyzoites and bradyzoites were screened by RT-PCR with gene-specific primers for the initiation and stop codons of TgAaaH1&2. The screen resulted in a PCR product of the expected size and sequence confirming the predicted coding sequence (Fig. 5A). RNA from both genes was detectable in both life cycle stages, although these cultures may have contained some mixture of stages. In addition, expression of *T. gondii* tyrosine hydroxylase at the protein level was confirmed by Western blotting. A single band of a 64kDa protein was observed from tachyzoite and 6-day post pH shock bradyzoite cultures of parasite lysate probed with tyrosine hydroxylase antibody (Fig. 5B). Importantly, there were no bands on the gel migrating at the size of human tyrosine hydroxylase (Mr 58.6 kD) in the infected human foreskin fibroblasts (HFF). This was also confirmed by RT-PCR experiments with primers directed at human TyrH (data not shown). The high level of similarity between the two proteins could not be distinguished by Western blotting. The protein was principally found in the insoluble fraction of the parasite lysate; in contrast to all other described aromatic amino acid hydroxylases that are soluble proteins found in the cytosol. This is consistent with the enzyme passing through the ER and Golgi for processing prior to secretion. Immunostaining of parasitized fibroblasts containing tachyzoites and bradyzoites exhibit staining of the parasite membrane and parasitophorous vacuole (PV) but no visible staining of organelles consistent with secretion (to be published elsewhere).

**Figure 6:**
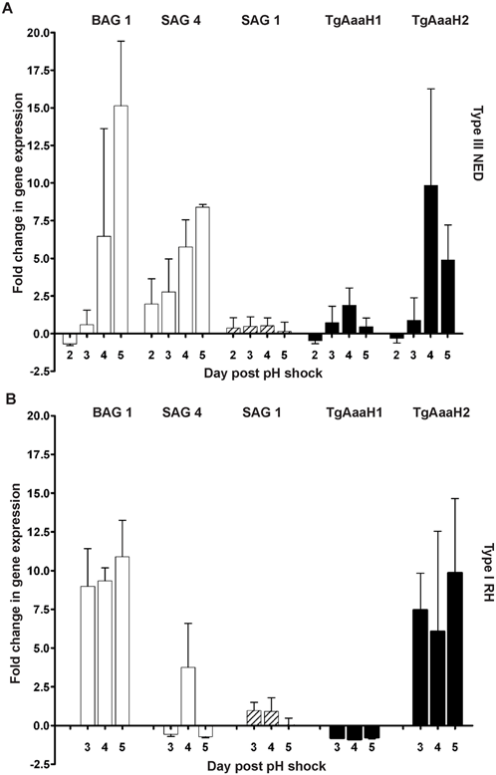
Stage specific mRNA expression of TgAaaH 1&2 during the tachyzoite to bradyzoite switch. Quantitative RT-PCR showing stage specific mRNA expression of TgAaaH 1&2 (black bars) in NED type III strain (A) and RH type I strain (B) parasites. Expression of SAG1 tachyzoite specific marker and BAG 1/SAG 4 bradyzoite specific marker genes (dashed and white bars respectively) was also followed to confirm differentiation to bradyzoites. This analysis shown here is relative to the housekeeping gene, GAPDH. Similar results are observed in analysis relative to actin (shown in supplementary data). Error bars are standard error of the mean (SEM) between flask replicates. Data was collected from day 2–5 for the NED strain, and days 3–5 for RH as preliminary experiments showed no change in gene expression at day 2 for RH.

Gene expression of mRNA encoding TgAaaH1&2 during pH shock induced stage conversion from the tachyzoite to the bradyzoite life cycle stages was monitored. Steady state levels of mRNA were measured by quantitative RT-PCR (Fig. 6 and supplemental Data S1). Differentiation was monitored using genetic markers (BAG1, SAG1, SAG4) (28–30) and by scoring changes in parasitemia and number of parasites per vacuole (data not shown). Both the avirulent type III strain NED, which forms bradyzoites readily in culture (31) and the type I strain RH were induced via pH shock (32). Expression of bradyzoite specific genes, BAG1 and SAG4 increased in both strains while expression of the tachyzoite specific gene SAG1 decreased post-induction.

TgAaaH1 mRNA was detectable in all stages whereas TgAaaH2 expression was induced during bradyzoite differentiation (Fig 6). In cultures of tachyzoites there are similar levels of TgAaaH1 and TgAaaH2. Levels of TgAaaH2 mRNA increased ten-fold and four-fold in NED and RH strains, respectively, whereas the levels of TgAaaH1 mRNA remained unchanged (Fig. 6). This observation of differential expression has been confirmed by recent microarray experiments (John Wooton, personal communication). Consistent with differences in switching in the strains, we observed a greater induction of TgAaaH2 and bradyzoite specific genes in the avirulent type III strain than the virulent type I strain. Therefore the parasite has increased expression of its tyrosine hydroxylase during brain and muscle cyst-forming stages of infection.

## Discussion

In this paper, we describe the characterisation of a bifunctional tyrosine hydroxylase from a protozoan parasite. *T. gondii* possesses two nearly identical genes that exhibit very similar kinetic properties yet differ in their developmental regulation of expression. They may have different biological roles. Intriguingly for a metabolic enzyme higher levels are expressed during bradyzoite differentiation, the stage of the life cycle with low metabolic activity. Unlike metazoan enzymes of this class, the *T. gondii* enzymes are bifunctional as they are able to utilize tyrosine and phenylalanine. Of interest, this enzyme can generate the tyrosine for l-DOPA synthesis by metabolizing phenylalanine to tyrosine. This was observed to some degree in experiments with tritiated phenylalanine based on analysis by HPLC (Fig. 4A). This gene was not found in any other apicomplexan except the closely related *N. caninum* in bioinformatics searches of available genome sequences. It is possible that this enzyme represents an unique feature of the tissue cyst forming apicomplexan parasites.

The *T. gondii* tyrosine hydroxylase has two domains. The C-terminal domain is responsible for catalysis and the N-terminal domain determines the substrate specificity. The C-terminal domain mutants Δ239TgAaaH 1&2 retained full activity. Indeed, the v_max_ was slightly higher for the two deletion mutants. This is similar to the results with other eukaryotic aromatic amino acid hydroxylases. The two-fold and three-fold preference for tyrosine versus phenylalanine in TgAaaH 1&2, respectively, is lost in the N-terminal deletion mutants. The K_Mphe_∶K_Mtyr_ ratio is two fold lower with removal of the regulatory domain whereas the V_max(phe)_∶V_max(tyr)_ ratio remains essentially unchanged (Fig. 4C). The N-terminal domain greatly contributes to substrate specificity of aromatic amino acid hydroxylases. N-terminal deletion mutants of rat TyrH and PheH (phenylalanine hydroxylase) expressed proteins with K_M_ values of 0.97 and 4.33, respectively, greatly reduced from the full length proteins (20).

Regulation of the activity of *T. gondii* tyrosine hydroxylase is likely to be complex. All described eukaryotic aromatic amino acid hydroxylases are phosphoproteins whose activity is regulated by a series of serine-threonine kinases. There is a complex network of phosphorylation of the N-terminal domain of eukaryotic tyrosine hydroxylase that regulate activity *in vivo*. The *T. gondii* orthologs have an extended N-terminal domain following the signal peptide that contains several potential phosphorylation sites (Fig. 1). The region is serine rich, a feature typical of phosphoproteins and *T. gondii* transit peptides (33,34). There is potential for phosphorylation by host cell kinases such as those that regulate human tyrosine hydroxylase. There is also potential for regulation by *T. gondii* proteins that are released into the host including the ROP kinases and the *T. gondii* 14-3-3 (35). Increased levels of host cell 14-3-3, a well-described activator of tyrosine hydroxylase, transcripts upon *T. gondii* infection have been detected, although decreased protein levels were observed in a proteomic study (9,10,36). Definition of the role of the N-terminal domain in regulation of the *T. gondii* tyrosine hydroxylase and of the parasite and host proteins involved awaits further experimentation.

*T. gondii* tyrosine hydroxylase requires the co-factor biopterin for catalytic activity (Fig. 4B), and can therefore be classed as a biopterin dependent aromatic amino acid hydroxylase. As relatively few enzymes require biopterin for activity, the discovery of a biopterin-dependent enzyme raises the issue of the possible sources of biopterin for this enzyme. The presence or absence of a biopterin synthetic pathway and its recycling are currently under investigation (37,38). *T. gondii* has been shown to encode a functional pterin-4a-carbinolamine dehydratase involved in recycling biopterin but dihydropterin reductase has not been detected (39). As the enzyme is secreted, the host may supply the biopterin cofactor. Indeed, human GTP cyclohydrolase, limiting in biopterin synthesis, has been found to be elevated in. *T gondii*-infected HFF cells (10).

The biological role(s) of a bifunctional tyrosine hydroxylase in *T. gondii* remain unclear. Expression of TgAaaH2 mRNA increases up to ten-fold in parasites during conversion from the fast-growing tachyzoite stage to the cyst-forming bradyzoite stage while TgAaaH1 mRNA is unchanged (Fig. 6). Indeed, TgAaaH2 expression may be strictly stage specific as tachyzoite cultures may contain a small proportion of bradyzoites from spontaneous conversion, as found in previous studies (40). Assessment of regulation at the protein level will require further experimentation to differentiate the two gene products. The regulation may be another example of differential expression of multiple isoenzyme forms during stage conversion similar to enolase (41) and lactate dehydrogenase (42), although in this case the enzyme is increased in bradyzoites.

The amino terminus of *T. gondii* tyrosine hydroxylase is similar to signal peptide motifs unlike other tyrosine and phenylalanine hydroxylases. The sequence is conserved among the two *T. gondii* tyrosine hydroxylase genes (Fig. 1). The signal peptide motif is also conserved in the *N. caninum* ortholog (unpublished results). It will be interesting to discover whether *S. neurona* has a similar hydroxylase. In initial experiments, immunostaining localizes the enzyme to the parasite membrane and parasitophorous vacuole and there is no staining of rhoptries or dense granules (preliminary observations). Confirmation of the PV location and the mechanism of trafficking of the hydroxylase into the PV require further experimentation.

There are several possible biological roles for a bifunctional *T. gondii* tyrosine hydroxylase. It is possible that the enzyme is required for supply of tyrosine for protein synthesis. Based on the higher substrate selectivity for tyrosine, synthesized tyrosine will be converted to l-DOPA (Fig. 4C). With this in mind, the question arises what is the role of l-DOPA synthesis by *T. gondii*. l-DOPA is the precursor to dopamine and increased levels of dopamine have been detected in the brains of infected rodents (43). *T. gondii* infects neuronal and glial cells and forms cysts during latent infection. This has led to the hypothesis that increased dopamine during infection is associated with the observed behavioural changes (6,44,45). It is plausible that the parasite tyrosine hydroxylase is responsible for the increased dopamine levels. Alternatively, l-DOPA may be involved in *T. gondii* tissue cyst wall formation; although the excreted oocyst of *Eimeria maxima* differs substantially from tissue cysts this related coccidian parasite has been found to contain l-DOPA in its oocyst wall glycproteins (46). l-DOPA has also been associated with oxidative damage in cells (47). Perhaps parasite generated l-DOPA increases reactive oxygen species (ROS) in host cells. The reasons for *T. gondii* to evolve stage specific expression of tyrosine hydroxylase remain unclear, but finding a dual function may indicate different biological roles are required for specific metabolic requirements in each life cycle stage.

## Materials and Methods

### Parasite strains and Reagents

*Toxoplasma gondii* strains used; virulent type I RH-YFP strain (48) and avirulent, cyst forming type III NED (a gift from Johan Lindh). All chemicals were purchased from Sigma, with the exception of 6-MPH_4_ from Schircks laboratories (Jona, Switzerland), Talon resin from Clontech and anti-tyrosine hydroxylase monoclonal antibody from Calbiochem (San Diego, CA).

### Parasite Culture

*Toxoplasma gondii* parasites were maintained in continuous passage in Hs27 human foreskin fibroblast (HFF) cell monolayers (ECACC number 94041901). Parasites were grown in DMEM supplemented with 10% foetal bovine serum (Invitrogen, Paisley, UK) and passaged between 4 and 15 times prior to bradyzoite induction. Bradyzoites were induced in culture using the high (pH 8.1) pH shock method (49). The number of parasitized cells, vacuoles and the number of parasites per vacuole were scored every 24 hours to monitor differentiation. Four parasitized monolayers were harvested every 24 hours by trypsinization and parasites purified by passage through a 21 gauge needle and washing in phosphate-buffered saline (PBS). Parasites were enumerated and pellets were resuspended in Qiagen RNeasy kit RTL buffer prior to RNA extraction. For Western blotting, parasites were harvested as in Roberts *et al.* (50). Proteins were separated by SDS-PAGE, transferred onto nitrocellulose membrane and probed with anti-rat-tyrosine hydroxylase monoclonal antibody.

### Prediction of the *T. gondii* tyrosine hydroxylase genes

The SHARKhunt search programme, part of the metaSHARK package for automated reconstruction of metabolic pathways, was used to predict enzymes encoded in the *T. gondii* genome (sequence freely provided to the community by the Welcome Trust Sanger Institute, Cambridge, UK and the Institute for Genomic Research (TIGR), USA) (15–17). SHARKhunt uses HMMER profile hidden Markov models (HMM's) based on the PRIAM library of polypeptide profiles (51) to search a set of DNA sequences (finished chromosomes, contigs or expressed sequence tags) for potential enzyme encoding genes (52). The current enzyme collection contains 2562 profiles, covering 1967 enzymatic functions as defined by E.C. number. Regions of DNA showing some similarities to these profiles are analysed in detail using GeneWise (53), which reconstructs a putative gene structure wherever possible. Finally a confidence score in the form of an E-value is calculated to represent the similarity between each predicted protein product and its corresponding profile model. Applying this software resulted in a set of genes whose sequences were compared with sequences of predicted signal peptide containing proteins (based on SignalP) in the *T. gondii* genome resource ToxoDB providing a list of potentially secreted proteins (www.toxodb.org, (18,19)). SHARKhunt predicted a tyrosine hydroxylase in a reconstruction of the pathway for *de novo* dopamine biosynthesis from the *T. gondii* genome that was subsequently found to be encoded by two genes via ToxoDB. These genes are mapped to different locations on chromosome V in the *T. gondii* genome at ToxoDB. The genomes of the apicomplexan parasites *Neospora caninum* (Wellcome Trust Sanger Centre and the University of Liverpool), *Plasmodium falciparum* (54), *Plasmodium vivax* (The Institute for Genomic Research), *Theileria annulata* (55), *Babesia bovis* (56), *Cryptosporidium parvum* (57) and *Eimeria tenella* (Wellcome Trust Sanger Centre and BBSRC Institute for Animal Health) were also analysed for presence of an aromatic amino acid hydroxylase.

Sequence alignments were created using Muscle multiple alignment software (58). The signal peptide is predicted by SignalP (59) to reside between positions 1 and 24 based on eukaryotic networks prediction and HMM of eukaryotic models prediction (60).

### Quantitative RT-PCR

Total RNA was extracted from purified *T. gondii* cell pellets using Qiagen RNeasy kit according to the manufacturers' instructions, and cDNA synthesis performed using Superscript II reverse transcriptase (Invitrogen). Quantity and quality of the RNA was monitored by spectrophotometry and by gel electrophoresis. To confirm stage conversion to bradyzoites, the RNA expression of three marker genes was also followed throughout the experiment. These were SAG 1 (primer sequences 5′-CGACAGCCGCGGTCATTCTC-3′ and 5′-CGACAGCCGCGGTCATTCTC-3′) (28), SAG 4, (primer sequences 5′-TGGACCTACGATTTCAAGAAGGC-3′ and 5′-GCTGCGAGCTCGACGGGCTCATC-3′) (29) and BAG 1 (primer sequences 5′-TCCGCCGGGAGCTTGTCCACC-3′ and 5′-GCAAGTCAGCCAAAATAATCA-3′) (30). Primers were designed to detect two housekeeping genes, actin (primer sequences 5′-CGAGCTGGTCAGTTCCTCAT-3′ and 5′-CATCGGGCAATTCATAGGAC-3′) and glyceraldehyde 3-phosphate dehydrogenase (GAPDH) (primer sequences 5′-GTATTGGCCGTCTGGTGTTC-3′ and 5′-CGTGGACCGAGTCGTATCTC-3′) for standardization. Quantitative PCR was carried out using Abgene SYBR green master mix (Rockford, IL) in a Bio-Rad I-cycler (Hercules, CA) for 40 cycles. Melt curve analysis was used to confirm the absence of primer-dimer formation or genomic DNA (Bio-Rad I-cycler QI software version 3.0). All expression profiles were standardised against parasite GAPDH and parasite Actin as housekeeping genes. All primers were specific to parasite cDNA and did not amplify HFF cDNA.

### PCR amplification and open reading frame confirmation of TgAaaH 1 & 2

cDNA from RH and NED parasites was synthesized as above. For both genes 3′ RACE was used to confirm the 3′ UTR terminus, and walking up the gene with primers confirmed the 5′ transcription initiation sites (Fig. 2). The second MET codon within the ORF (open reading frame) of TgAaaH1 is assumed to be the translation start site due to conservation with TgAaaH2 and a conserved predicted signal peptide. This is also found in the predicted coding region of the *N. caninum* gene. PCR amplification using primers within the 5′ and 3′UTR of both genes allowed specific amplification of each transcript. Both genes were subcloned into pGEM-T Easy vector (Promega, Madison, WI) for sequencing. There were no coding differences between the RH and NED genes.

### Expression and purification of recombinant proteins

Restriction sites for *Pst I* and *Not 1* were added to the termini of both full length ORFs by PCR using Phusion polymerase (Invitrogen) from the pGEM-T constructs. Primers were: forward primer 5′-CTGCAGATGTGGCAGGCCATTTTCGCTG-3′, reverse primer (TgAaaH1) 5′-GCGGCCGCTAGAACCTGAGGGAAACGGG-3′ and reverse primer (TgAaaH2) 5′-GCGGCCGCTAGATCTTGAGGGAGACGGG-3′. The PCR products were again sub-cloned into pGEM-T easy vector (Promega), digested with *Pst I* and *Not I* restriction enzymes and inserted into pET 45b expression vector (Novagen, Madison, WI) with Promega ligase. Vectors containing the correct insert were confirmed by sequencing and double digest with *Pst I/Not I* and with *Bam HI* only. These constructs were named Apo-TgAaaH1 and Apo-TgAaaH2.

Deletion constructs were generated by PCR with Phusion polymerase using primers beginning at residue 23 (5′- GGTTCTGCAGGCTGTCCCCCAAGAATC-3′) to residue 239 (5′-CTGCAGGTCCCGTGGTTCCCTCGGTCT-3′) from both TgAaaH 1 and TgAaaH2 cloned in pGEM-T plasmids, generating a terminal *Pst1* site and an in-frame start codon. The PCR products were cloned into pGEM-T easy before sub-cloning into pET 45b as above. Deletion of the first 23 residues removed the region predicted to be a signal peptide (Fig. 2), these were then referred to as TgAaaH 1&2. Deletion of the first 239 residues results in constructs beginning at the VPWFPR motif at the start of the putative catalytic domain (20, 61, 62), these were then named Δ239TgAaaH 1 & 2. All constructs were sequenced in pET 45b prior to expression (Fig. 3).

All pET 45b constructs were transformed by electroporation into BL21 GOLD (DE3) pLysS *E. coli* (Novagen). Cultures were grown for ∼4 hrs at 37°C in TB media supplemented with 10 µM ferrous ammonium sulphate to an OD_600_ of ∼0.8, induced with 0.1 mM IPTG and shaken for 20 hrs at 18°C (TgAaaH) or 28°C (Δ239TgAaaH). Cultures were harvested by centrifugation at 10,000×g for 5 min and pellets stored at −70°C overnight. All cell pellets were resuspended in 50 mM Tris-HCL pH 7.5, plus 0.2% Triton-X 100 and EDTA-free protease inhibitors (Roche, Nutley, NJ). These were sonicated for 30 sec with 30 sec rest three times on ice. Extracts were spun at 20,000×g for 20 min at 4°C and the soluble fraction removed. This was repeated two times with ten cycles of 30 s sonication/30 s rest on ice. The resulting soluble fraction was passed over a Talon affinity resin column (Clontech) and eluted with 500 mM imidazole. The imidazole was removed by dialysis for 2 hrs at 4°C against 50 mM Tris-HCL pH 7.5, plus 0.2% Triton-X100. Coomasie-stained SDS-PAGE electrophoresis and Western blotting with anti-rat-tyrosine hydroxylase monoclonal antibody and anti-His-tag antibody assessed the purity of each protein. As tyrosine hydroxylases contain an iron atom embedded in the structure that is required for catalytic activity, we increased the Fe^2+^ concentration of the expression media by the addition of ferrous ammonium sulphate to ensure that all the recombinant enzyme would contain Fe^2+^ (63–65). The additional iron increased the percentage of soluble protein suggesting that the presence of the central iron atom may be required for correct folding in this expression system. The addition of 0.2% Triton-X 100 and low salt concentrations also enhanced the solubility as seen with previous recombinant tyrosine hydroxylase enzymes (66).

Constructs for the expression of rat tyrosine hydroxylase (rat-WT-TyrH) and the catalytic domain of rat phenylalanine hydroxylase (rat-Δ117PheH) in pET 23d, were a kind gift from Paul Fitzpatrick. Both proteins were expressed without a tag and enriched by step-wise ammonium sulphate precipitation (61).

### Biochemical assays

Assays were developed and standardized using recombinant proteins from expression of rat-WT-TyrH and rat-Δ117PheH. Protein concentration was determined using the Bradford assay (67). All assays were initiated by the addition of 10 µg enzyme. As the reproducibility of biopterin-dependent enzyme kinetics are greatly affected by the instability of the biopterin cofactor, 6-MPH_4_ was used and checked prior to assay by assessing the 240–300 nm spectrum. Assays were 10 min as biopterins are unstable in air and prolonged incubation with BH_4_ ((6R)-tetrahydropterin) will inactivate these enzymes (68, 69). The hydroxylation of tyrosine was measured by the method of Fitzpatrick (25) with the modifications of Royo *et al.* (26). Conditions were 25°C, pH 7.5, 10 mM HEPES, 1 mM DTT, 100 µg/ml catalase and 10 µM ferrous ammonium sulphate. Assays were terminated at 10 min by the addition of 15 µl 50% HCL, 200 µl 12.5% sodium nitrite and 200 µl 12.5%. NaOH (70 µl 3 M) was then added to each reaction and the absorbance at 500 nm read precisely 10 sec later. All assays were initiated by the addition of the enzyme. Additionally, the hydroxylation of phenylalanine was assayed by direct measurement of the production of tyrosine at 275 nm according to the method of Pember *et al.* (70) with the modifications of Daubner *et al.* (71). Conditions were 25°C, pH 7.5, 80 mM HEPES, 5 mM DTT, 60 µg/ml catalase and 10 µM ferrous ammonium sulphate. Tyrosine and dopa production was also detected by reverse phase HPLC of tritiated products. L-[2,3,4,5,6-^3^H]phenylalanine (25 µCi; final chemical concentration 1 µM) or 25 µCi L-[2,3,5,6-^3^H]tyrosine (final chemical concentration 1.25 µM) (GE Healthcare) was added to 10 µg recombinant protein in 200 µl reaction volumes. Reaction conditions were 500 µM 6-MPH_4_, 50 mM HEPES, 20% glycerol, 0.25% Triton-X 100 and EDTA free protease inhibitors (Roche) at pH 7.5. Reactions were initiated by the addition of enzyme and allowed to proceed for 4 hrs at 37°C. Reactions were stopped by freezing in liquid nitrogen and stored at −80°C prior to HPLC analysis.

In preparation for HPLC 50 µl of each assay mix was added to 210 µl 0.1% TFA (trifluroacetic acid). To this was added 5 µl of 1 mg/ml dopa, tyrosine and phenylalanine as standards. Samples were spun at 13000×g in a bench top microfuge to pellet the precipitated protein. Products were separated by reverse phase HPLC on a Synergi 4u Hydro 80A column (150×4.60 mm, Phenomenex, Macclesfield, UK) with an elution gradient of 0–60% acetonitrile with 0.1% TFA over 20 min at a flow rate of 1 ml/min. Fractions (0.5 ml) were collected every 30 sec from 6 min-18 min for each sample. Each fraction was counted for 1 min in 3 ml liquid scintillant (Packcard, Minnesota, USA) on a Wallac 1450 Microbeta Trilux counter using default settings.

For kinetic calculations an assay measuring the oxidation of 6-MPH_4_ using a coupled reaction measuring the decrease in absorbance at 340 nm due to NADH oxidation (61) was used as this assay has been validated for use in several previous studies (20,25,66). Conditions were 32°C, pH 7.5, 10 µg purified protein, 50 mM HEPES, 100 µg/ml catalase, 500 µM NADH, 10 µM ferrous ammonium sulphate, 0.2 u/ml sheep liver dihydropteridine reductase. For K_mTyr_/K_mPhe_ measurements concentrations of amino acids were varied from 0–200 µM with 500 µM 6-MPH_4_. For K_m6-MPH4_ concentrations of 6-MPH_4_ were varied from 0–500 µM with 50 µM of each amino acid separately.

### Data analysis

Kinetic data was fitted to the Michaelis-Menten equation using Graphpad PRISM (San Diego, California).

## Supporting Information

Data S1 Supplemental Data S1. Stage specific mRNA expression of TgAaaH 1&2 during the tachyzoite to bradyzoite switch against actin as a housekeeping gene. Quantitative RT-PCR showing stage specific mRNA expression of TgAaaH 1&2 (black bars) in NED type III strain (A) and RH type I strain (B) parasites. Expression of SAG1 tachyzoite specific marker and BAG 1/SAG 4 bradyzoite specific marker genes (dashed and white bars respectively) was also followed to confirm differentiation to bradyzoites. The analysis shown here is relative to actin as a housekeeping control gene. Error bars are error between flask replicates. Data was collected from day 2–5 for the NED strain, and days 3–5 for RH as preliminary experiments showed no change in gene expression at day 2 for RH.
(22.42 MB TIF)
