# Supplementary figures and images for: A Unique Dual Activity Amino Acid Hydroxylase in Toxoplasma gondii
Source: PLoS One. 2009 Mar 11;4(3):e4801. doi: 10.1371/journal.pone.0004801 (PMC2653193; doi:10.1371/journal.pone.0004801)

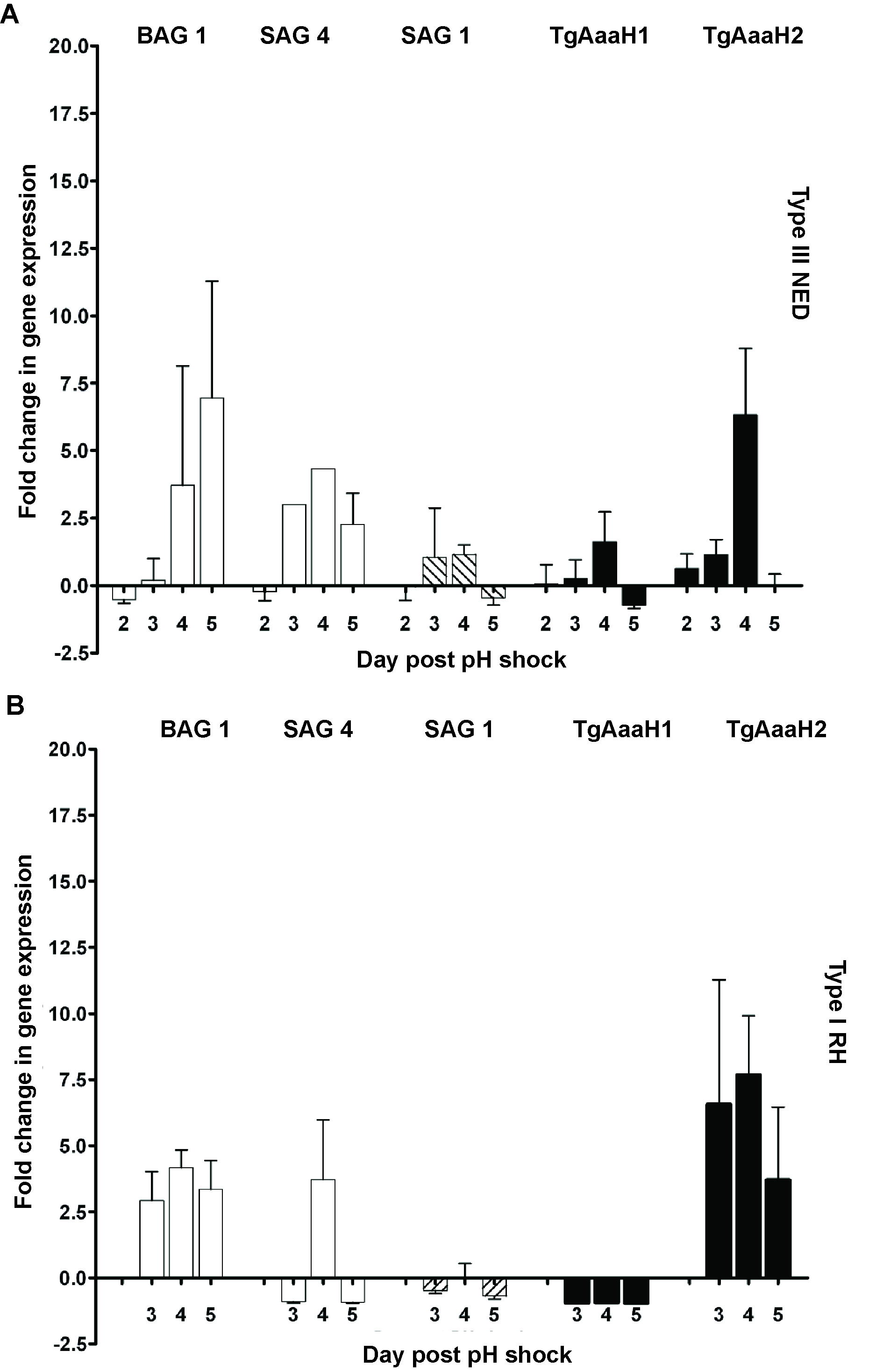

Supplement: Data S1 — Supplemental Data S1. Stage specific mRNA expression of TgAaaH 1&2 during the tachyzoite to bradyzoite switch against actin as a housekeeping gene. Quantitative RT-PCR showing stage specific mRNA expression of TgAaaH 1&2 (black bars) in NED type III strain (A) and RH type I strain (B) parasites. Expression of SAG1 tachyzoite specific marker and BAG 1/SAG 4 bradyzoite specific marker genes (dashed and white bars respectively) was also followed to confirm differentiation to bradyzoites. The analysis shown here is relative to actin as a housekeeping control gene. Error bars are error between flask replicates. Data was collected from day 2–5 for the NED strain, and days 3–5 for RH as preliminary experiments showed no change in gene expression at day 2 for RH. (22.42 MB TIF) [file pone.0004801.s001.tif]
